# Vaginal CO_2_ laser therapy for genitourinary syndrome in breast cancer survivors—VagLaser study protocol: a randomized blinded, placebo-controlled trial

**DOI:** 10.1186/s12885-023-11656-x

**Published:** 2023-11-29

**Authors:** Sine Jacobsen, Marianne Glavind-Kristensen, Anders Bonde Jensen, Axel Forman, Pinar Bor

**Affiliations:** 1 Department of Obstetrics and Gynaecology, Randers Regional Hospital, Randers, Denmark; 2 Department of Clinical Medicine, Aarhus University, Aarhus, Denmark; 3 Department of Obstetrics and Gynaecology, Aarhus University Hospital, Aarhus, Denmark; 4 Department of Oncology, Aarhus University Hospital, Aarhus, Denmark

**Keywords:** Vaginal laser, Genitourinary syndrome of menopause, Mamma cancer, Vaginal dryness

## Abstract

**Background:**

Vaginal CO_2_ laser therapy is a new treatment option for genitourinary syndrome of menopause. Its potential is particularly interesting in breast cancer survivors, where existing treatment options often are insufficient as hormonal treatment is problematic in these women. The objective of this study is to investigate the effectiveness of vaginal laser treatment for alleviation of genitourinary syndrome of menopause in breast cancer survivors treated with adjuvant endocrine therapy. The secondary objective is to explore the importance of repeated vaginal laser treatment and the long-term effects in this patient population.

**Methods:**

VagLaser consist of three sub-studies; a dose response study, a randomized, participant blinded, placebo-controlled study and a follow-up study. All studies include breast cancer survivors in adjuvant endocrine therapy, and are conducted at the Department of Obstetrics and Gynecology, Randers Regional Hospital, Denmark. The first participant was recruited on 16th of February 2023. Primary outcome is vaginal dryness. Secondary subjective outcomes are vaginal pain, itching, soreness, urinary symptoms and sexual function. Secondary objective outcomes are change in vaginal histology (punch biopsy), change in vaginal and urine microbiota, and change in vaginal pH.

**Discussion:**

More randomized controlled trials, with longer follow-up to explore the optimal treatment regimen and the number of repeat vaginal laser treatments for alleviation the symptoms of genitourinary syndrome of menopause in breast cancer survivors treated with endocrine adjuvant therapy, are needed. This study will be the first to investigate change in vaginal and urine microbiota during vaginal laser therapy in breast cancer survivors.

**Trial registration:**

ClinicalTrials.gov: NCT06007027 (registered 22 August, 2023).

Protocol version: Version 1, Date 13.11.2023.

## Item from the World Health Organization trial registration data set

**Table:** 

Data category	Information
Primary registry and trial identifying number	ClinicalTrials.gov: NCT06007027
Date of registration in primary registry	22 August, 2023
Primary sponsor	Pinar Bor
Secondary sponsors	Marianne Glavind-Kristensen and Anders Bonde Jensen
Contact for public queries	Sine Jacobsen, MD, Mobile + 4560244277, Email sinjac@rm.dk
Contact for scientific queries	Sine Jacobsen, MD Department of Obstetrics and Gynaecology, Randers Regional Hospital, Denmark Department of Clinical Medicine, Aarhus University, Denmark
Public title	Vaginal CO_2_ laser therapy for genitourinary syndrome in breast cancer survivors
Scientific title	Vaginal CO_2_ laser therapy for genitourinary syndrome in breast cancer survivors—A randomized blinded, placebo-controlled trial
Country of recruitment	Denmark
Health condition(s) or problem(s) studied	Genitourinary syndrome of menopause
Intervention(s)	Active comparator: SmartXIDE^2^V^2^LR, MonaLisa Touch, DEKA, Florence, Italy (Setting: dot power 30 W, dwell time 1000 μs, dot spacing 1000 μs and the smart stack parameter from 2–3) Placebo comparator: SmartXIDE^2^V^2^LR, MonaLisa Touch, DEKA, Florence, Italy (Setting: 0 W, dwell time 100 μs, dot spacing 2000 μs and the smart stack parameter from 1 to 1)
Key inclusion and exclusion criteria	Key inclusion criteria: Breast cancer survivor in endocrine therapy, age > 18 years, symptomatic genitourinary syndrome of menopause with vaginal discomfort and/or dyspareunia Key exclusion criteria: Use of non-hormonal/hormonal vaginal therapies (1 and 12 months prior to the baseline visit, respectively), treatment with Chemotherapy (6 months prior to the baseline visit)
Study type	Interventional Allocation: randomized Intervention model: parallel assignment Masking: blinded (participants) Primary purpose: reducing late effects of breast cancer treatment
Date of first enrolment	February 2023
Target sample size	90 participants
Recruitment status	Recruiting
Primary outcome	Vaginal dryness
Key secondary outcomes	Subjective outcomes are vaginal pain, itching, soreness, urinary symptoms and sexual function Objective outcomes are change in vaginal histology (punch biopsy), change in vaginal and urine microbiota and change in vaginal pH

## Background

Breast cancer (BC) is the most common type of cancer among women worldwide. Adjuvant endocrine therapy is offered to those 80% of the breast cancer patients with estrogen positive disease. Thus, pre- and peri-menopausal women are treated with tamoxifen, a selective estrogen receptor modulator (SERM) which will block the effect of estrogen, for 10 years if node positive and 5 years if node negative in the axilla. Post-menopausal women are treated with an aromatase inhibitor (AI) in five years, e.g., letrozole, which inhibits the production of estrogen. Notably, 50–75% of breast cancer survivors (BCS) experience one or more symptoms of genitourinary syndrome of menopause (GSM) [1], which is defined as a collection of signs and symptoms such as vaginal dryness, dyspareunia, irritation, urinary incontinence and urinary tract infections [2]. Vaginal treatment with estrogen can alleviates GSM symptoms but significant absorption through the vaginal epithelium has been reported [3]. This leads to concerns about the risk of cancer recurrence [4] and many BCS want to avoid estrogen-containing treatment. An effective, non-hormonal, and safe treatment of GSM in women diagnosed with BC is lacking today.

Fractional CO_2_ laser removes layers of skin tissue and causes regeneration of scar tissue. The treatment is given as arrays of small laser beams creating a matrix of microscopic epithelial and sub-epithelial thermal necrosis. This induces a wound-healing cascade with formation of elastin- and collagen fibers resulting in tissue remodeling [5, 6]. Furthermore, laser therapy may improve proliferation of vaginal epithelial cells and glycogen production, which is essential for the growth of vaginal lactobacilli. This results in increased lactobacillus flora and a lowering of vaginal pH needed to maintain vaginal health and avoid local inflammation [7]. The prevalence of lactobacilli has been reported to change from 30 to 79% in postmenopausal women after vaginal laser treatment [7]. Vagina and bladder share the same commensal bacteria. A high number of lactobacilli is also important in maintaining bladder health by lowering pH which inhibits growth of many uropathogens and prevents attachment of uropathogens to uroepithelial cells [8–10]. Overall, high occurrence of lactobacilli prevents infection. Vaginal laser therapy is a safe procedure, and no serious adverse events have been reported [11].

Previous studies showed improvements of GSM symptoms by vaginal laser treatment in women with spontaneous menopause and iatrogenic menopause [12–15]. However, iatrogenic menopause after breast cancer treatment with endocrine therapy represents a unique endocrine situation. Very limited studies have investigated the optimal treatment regime and the number of repeat laser treatments to obtain comparable effects of vaginal laser treatment on GSM in BSC. A recently published review by Mortensen et al. [11] concluded that larger long-term and high-quality RCTs are needed within this field before vaginal laser can be considered a routine treatment for GSM in BSC.

### Objectives

The primary objective is to investigate the effectiveness of vaginal laser treatment for alleviation of vaginal dryness which is the cardinal symptom of GSM symptoms in BCS treated with endocrine adjuvant therapy in the placebo-controlled study. Vaginal pain, itching, soreness, urinary symptoms and sexual function will be accessed as secondary subjective outcomes and change in vaginal histology (punch biopsy), change in vaginal and urine microbiota and change in vaginal pH will be accessed as secondary objective outcomes. This project also evaluates the long-term effects, including provide information on the optimal number of repeat vaginal laser sessions and effectiveness of three versus four versus five vaginal laser treatment in this patient population.

## Methods

The VagLaser trial is a single-center study containing three sub-studies:

I. A dose–response study exploring the optimal number of laser treatments needed to achieve an effect on GSM symptoms in BCS. A total of 30 participants will be included for a maximum of five treatments of vaginal laser therapy at 4–6 weeks' intervals.
II. A randomized, participant blinded, placebo-controlled study to compare vaginal laser therapy with sham laser therapy in 60 BCS, with a fixed number of treatments. The number of vaginal laser treatments depends on results obtained from study I.
III. A one-year follow up study on participants from study II. The participants from the active laser group will be offered a single "booster" treatment.

### Site

Department of Obstetrics and Gynecology, Randers Regional Hospital, Denmark.

### Study period

The first participant was recruited the 16th of February 2023. The anticipated date of the last follow up is June 2025.

### Approval of the study protocol

The study has been approved by the Danish Data Protection Agency (1–16-02–327-22) and by the Danish Ethical Committee (1–10-72–183-22).

### Participants

The VagLaser trial includes breast cancer survivors in endocrine treatment (SERM or AI) with GSM symptoms. Inclusion and exclusion criteria for participation (Table 1) are verified before starting the first vaginal laser application.

**Table 1:** Criteria for inclusion and exclusion in the study

Inclusion criteria:	Exclusion criteria:
Age > 18 years	Pelvic organ prolapse ≥ stage 2 according to the Pelvic Organ Prolapse Quantification (POP-Q) staging system
Breast cancer survivor in endocrine therapy	Use of non-hormonal vaginal therapies (1 month prior to the baseline visit) and use of hormonal vaginal therapy (12 months prior to the baseline visit)
Symptomatic GDM with vaginal discomfort and/or dyspareunia	Treatment with chemotherapy (6 months prior to the baseline visit)
Able to read and understand Danish	Acute urinary tract infection or active genital infection
Able to give written informed consent	History of vaginal reconstructive surgery

Participants are recruited through Danish Breast Cancer Organization, Danish Organization for sequelae after cancer, social media (e.g., Facebook, Instagram), from Department of Obstetrics and Gynecology, Randers Regional Hospital, Department of Obstetrics and Gynecology, Aarhus University Hospital, Department of Oncology at Aarhus University Hospital, Department of Oncology at Aalborg University Hospital, Department of Oncology at Vejle Regional Hospital, Denmark.

## Vaginal CO_2_ laser protocol

### Active treatment

Included patients are treated intravaginally with the fractional microablative CO_2_ laser system (SmartXIDE^2^V^2^LR, MonaLisa Touch, DEKA, Florence, Italy), using the following settings: dot power 30 W, dwell time 1000 μs, dot spacing 1000 μs and the smart stack parameter from 2 to 3. At the level of the vaginal introitus, the dot power decreases to 20 Watt. The procedure is performed in the outpatient clinic and do not require any specific preparation (e.g. analgesia/anesthesia).

### Sham procedure used in the randomized study

Included patients are treated intravaginally with the fractional microablative CO_2_ laser system (SmartXIDE^2^V^2^LR, Monalisa Touch, DEKA, Florence, Italy), using the following settings: dot power 0.5 W, dwell time 100 μs, dot spacing 2000 μs and the smart stack parameter from 1 to 1. This procedure is identical to the treatment procedure and even the noise involved with firing the laser is maintained allowing blinding of the participants.

## Prohibited concomitant medications

The use of oral or vaginal hormonal therapies (e.g. Vagifem) is not permitted for the participants during the study period. If needed, participants may use a specific 92% rich fat cream ("Dr. Warming Critical Care") in the intimate area during the study period.

## Outcome measures

### Primary outcome

#### Vaginal dryness

Participant are asked to complete the 10-cm visual analog scale (VAS) ranging for 0 to 10, with higher score indicating worse vaginal dryness.

- Study I: Primary outcome is difference of vaginal dryness between baseline visit, and after third, fourth and fifth laser treatment.
- Study II: Primary outcome is difference in vaginal dryness four weeks after the last treatment between the laser group and the sham group.
- Study III: Primary outcome is vaginal dryness one year after the last laser treatment. Secondary outcome is the effect of one booster treatment on vaginal dryness.

### Subjective secondary outcomes

Vaginal pain, itching and soreness, evaluated with VAS for vaginal pain, itching, soreness.

#### Sexual function

Participant are asked to complete the questionnaire Female Sexual Function Index (FSFI) for sexual function parameters; desire, arousal, lubrication, orgasm, satisfaction and pain (range 2–36, the threshold of 26.55 indicates sexual dysfunction) [16] and Sexual complaint screener – women (SCS-W) questionnaire addressing all domains of sexual dysfunction (range 0–60, higher score indicate increased symptom severity [17].

#### Urinary symptoms

Evaluated by the questionnaire Urogenital Distress Inventory (UDI-6); irritative symptoms, obstructive/discomfort and stress symptoms (range 0–18, higher score indicate higher disability) [18] and International Consultation on Incontinence Questionnaire Female Urinary Tract Symptoms Sex (ICIQ-FLUTSsex) for evaluation of female sexual matters associated with their lower urinary tract symptoms (range 0–14, higher values indicating increased symptom severity) [19].

#### Vaginal health index (VHI)

A gynecologic examination is performed by an experienced gynecologist at the baseline visit and at each follow-up visits using the VHI. VHI includes subjective scoring of moisture, fluid volume, epithelial integrity and elasticity and objective scoring of pH (VHI ranges 5–25, higher VHI scores indicate better health).

### Objective secondary outcomes

#### Change in vaginal pH

Vaginal pH is measured using pH-Indicator strips.

#### Change in vaginal histology

A punch biopsy of 4*4 mm is taken from the lateral left vaginal wall at the baseline visit and from the lateral right vaginal wall at the follow-up visit. The biopsy is immediately formalin fixated and then processed for light microscopy. Some sections are stained using haematoxylin and eosin (H&E), and others with Masson's trichrome stain and immunohistochemistry test. The biopsies are evaluated by a specialized gynecological pathologist at The Department of Pathology Randers Regional Hospital, reporting on the changes in the vaginal histology.

#### Change in vaginal and urine microbiota

One polyester swap (FLOQSwab) is used to sample mid-vagina for microbial analysis and immediately been placed at – 80 °C until further processing. Urine is collected in a 50 mL collection tube using the clean catch method for women. Prior to voiding, the urethral meatus/urinary opening is cleaned with sterile water. Afterwards aliquoted into 10 mL fractions and immediately been placed at – 80 °C until further processing.

DNA is isolated from samples using standard DNA isolation kits and microbiota composition determined using 16S rRNA gene sequencing, which are performed at Centre for Clinical Research, North Denmark Regional Hospital. Microbiota composition is compared between groups and over time based on alpha- and beta-diversity measures, as well as changes in relative abundances of individual bacteria.

### Data collection

Data are collected electronically and stored in Research Electronic Data Capture (REDCap, Aarhus University), which is a secure online database. Demographic characteristics are collected from women included at baseline visit as follows: age, BMI, smoking, parities, age at menopause, years since breast cancer diagnosis, years since start at endocrine therapy, type of endocrine therapy.

- Study I: Symptom data are collected at baseline, after each treatment visit and 6 months after the initial treatment. Vaginal biopsy is collected at baseline visit and 6 months after the initial treatment. Vaginal and urine microbiota are collected at baseline visit, at each treatment visit, and 6 months after initial treatment (Table 2).
- Study II: Symptom data, vaginal biopsy, vaginal and urine microbiota are collected at baseline visit and one month after the last treatment (Table 3).
- Study III: Symptom data, vaginal biopsy, vaginal and urine microbiota are collected at one year follow-up visit which is scheduled one year after the completion of the last treatment in the study II, and one month after the single "booster" treatment.

**Table 2:** The timeline of the treatment protocol for study I is summarized

	**Baseline**	**Treatment no. 1**	**Treatment no. 2**	**Treatment no. 3**	**Treatment no. 4**	**Treatment no.5**	**4–6 weeks after 5.treatment**
**Timepoint (weeks/months)**	0 weeks	4 weeks	8 weeks	12 weeks	16 weeks	20 weeks	6 months
**Eligibility criteria**	x						
**Informed consent**	x						
**Information from the patient record**	x						
**Questionnaires**	x				x	x	x
**Gynaecological examination**	x		x	x	x	x	x
**Vaginal biopsy**	x						x
**Vaginal and urine Microbiota**	x		x	x	x	x	x

**Table 3:** The timeline of the treatment protocol for study II is summarized

	**Baseline**	**Treatment no. 1**	**Treatment no. 2**	**Treatment no.3**	**Treatment no.4/no. 5**	**4–6 weeks after the last treatment**
**Timepoint (weeks/months)**	0	4 weeks	8 weeks	12 weeks	-	4 months
**Eligibility criteria**	x					
**Informed consent**	x					
**Information from the patient record**	x					
**Questionnaires**	x					x
**Gynaecological examination**	x					x
**Vaginal biopsy**	x					x
**Vaginal and urine Microbiota**	x			x	x	x

### Randomization, blinding and informed consent

The women are e informed about the trial when they contact principal investigator. After signing of informed consent to participation, women are randomized in a 1:1 ratio to either laser group or sham group using an Internet-based randomization-program (REDCap, Aarhus University). Women are stratified by age, duration of prior endocrine treatment, and type of endocrine treatment. The participants in study II will be blinded to treatment allocation until the study has been completed.

### Side effects and adverse event

Vaginal laser therapy is associated with a low risk of complications. Most patients (97%) experience almost no or only mild discomfort in relation to the treatment [20]. The mild immediate symptoms after vaginal laser therapy are seen within the first two days and include burning sensation, itching, bruising and swelling [21]. The investigator will report to the Danish research ethics committees all suspected unexpected serious adverse reactions/events as soon as possible.

### Power calculation and statistical analysis

Considering the VAS score ranging for 0 to 10 for vaginal dryness as the primary outcome. In study one the sample size was calculated based on an expected mean 30% improvement on the VAS score for vaginal dryness from the third treatment compared to the fifth treatment. Aiming for a power of 90% and an alpha of 0.05 and a standard deviation of 2.9 was adopted [22], a sample size of 23 women were required. With an estimate loss of 20% participants during the five laser treatments, a total of 30 women will be included in study I. In study two the sample size was calculated based on an expected 30% improvement on the VAS score for vaginal dryness in the active laser group compared to the sham laser group. To achieve a power of 90% and an alpha of 0.05 with a standard deviation of 2.9 [22] it was determined that [22], a sample size of 21 women in each group was required. With an estimate loss of 20% participants during the second study, a total of 52 women will be included in study II, 26 in each group.

Statistical analyses will be performed using STATA. If the data followed a Gaussian distribution, the Student’s t-test will be used to test for differences between variable means. Otherwise, Mann Whitney's test will be used. For ordinal data from the questionnaires, Pearson’s χ^2^ and linear-by-linear tests will be performed. Paired test with one-way ANOVA will be used to evaluate change over time. Groups variables will be tested for change over time with two-way ANOVA (repeated measurements). A 2-sided *P*-value < 0.05 will be chosen as the level of statistically significance. Data are given as mean ± SD if they followed a Gaussian distribution; otherwise, median (range) are indicated.

## Discussion

One of the main problems among BCS in endocrine therapy are symptoms of GSM, and the fact that untreated GSM worsens over time exacerbate the problems further [23]. Local estrogen treatment seems to be safe in most BCS, but a large number of BCS are concerned about risk of cancer relapse and want to avoid any kind of estrogen-containing treatment. Thus, an alternative non-hormonal treatment for GSM in BCS is much needed. A recent published study by Mension et al. [24] found an overall improvement after vaginal laser therapy in BCS, but no statistically significant differences were observed in the active vaginal laser therapy group compared to the sham laser therapy group. In conclusion more RCTs with longer follow-up to explore the optimal treatment regime and the number of repeat vaginal laser treatments for alleviation of GSM symptoms in BCS treated with endocrine adjuvant therapy, are needed. Furthermore, this study will be the first to investigate change in vaginal and urine microbiota during vaginal laser therapy in BCS.

### Ancillary and Post-Trial Care

Participants that are enrolled into the study are covered by the Danish public patient compensation scheme, if unforeseen late effects of the treatment are seen.

## Data Availability

The trial is ongoing. When the primary data from the trial are published the data will become publicly available.

## Abbreviations

AI: Aromatase inhibitor
BCS: Breast cancer survivors
GSM: Genitourinary syndrome of menopause
SERM: Selective estrogen receptor modulator
